# Wild-type S100A3 and S100A13 restore calcium homeostasis and mitigate mitochondrial dysregulation in pulmonary fibrosis patient-derived cells

**DOI:** 10.3389/fcell.2023.1282868

**Published:** 2023-11-30

**Authors:** Eid A. Al-Mutairy, Somaya Al Qattan, Mohammed Khalid, Azizah A. Al-Enazi, Maher M. Al-Saif, Faiqa Imtiaz, Khushnooda Ramzan, Vineesh Raveendran, Ayodele Alaiya, Brian F. Meyer, Sergei P. Atamas, Kate S. Collison, Khalid S. Khabar, Jeffrey D. Hasday, Futwan Al-Mohanna

**Affiliations:** ^1^ Department of Cell Biology, King Faisal Specialist Hospital and Research Centre, Riyadh, Saudi Arabia; ^2^ Department of Medicine, King Faisal Specialist Hospital and Research Centre, Riyadh, Saudi Arabia; ^3^ BioMolecular Medicine, King Faisal Specialist Hospital and Research Centre, Riyadh, Saudi Arabia; ^4^ Clinical Genomics, Center of Genomic Medicine, King Faisal Specialist Hospital and Research Centre, Riyadh, Saudi Arabia; ^5^ Stem Cell Therapy Program, King Faisal Specialist Hospital and Research Centre, Riyadh, Saudi Arabia; ^6^ University of Maryland School of Medicine, Baltimore, MD, United States; ^7^ Baltimore VA Medical Center, Baltimore, MD, United States

**Keywords:** lung, fibrosis, calcium, mitochondria, S100A3, S100A13

## Abstract

Patients with digenic S100A3 and S100A13 mutations exhibited an atypical and progressive interstitial pulmonary fibrosis, with impaired intracellular calcium homeostasis and mitochondrial dysfunction. Here we provide direct evidence of a causative effect of the mutation on receptor mediated calcium signaling and calcium store responses in control cells transfected with mutant S100A3 and mutant S100A13. We demonstrate that the mutations lead to increased mitochondrial mass and hyperpolarization, both of which were reversed by transfecting patient-derived cells with the wild type S100A3 and S100A13, or extracellular treatment with the recombinant proteins. In addition, we demonstrate increased secretion of inflammatory mediators in patient-derived cells and in control cells transfected with the mutant-encoding constructs. These findings indicate that treatment of patients’ cells with recombinant S100A3 and S100A13 proteins is sufficient to normalize most of cellular responses, and may therefore suggest the use of these recombinant proteins in the treatment of this devastating disease.

## Introduction

Progressive loss of lung function leading to respiratory failure is the major consequence of irreversible pulmonary fibrosis (PF). Idiopathic pulmonary fibrosis (IPF) is the most common of the interstitial lung diseases (ILD) with poor prognosis (Maher et al., 2021; Zhang et al., 2021; Pitre et al., 2022). The disease affects around three million patients worldwide (Glassberg, 2019) with an incidence of 3–9 cases per 100,000 per year (Hutchinson et al., 2015) or 2–30 cases per 100,000 per year (She et al., 2021), depending on the population, with minimal treatment options (Raghu et al., 2015; Martinez et al., 2017; Rosas et al., 2018; She et al., 2021).

Although the majority of IPF cases seems sporadic a number of risk factors have emerged. These include cigarette smoking (Baumgartner et al., 1997), environmental and occupational factors including wood or metal dust and pesticides (Seo et al., 2019; Park et al., 2021), certain medications (Daba et al., 2004) and chronic viral infection (Sheng et al., 2020). Typically genetic predisposition has been linked to ILD in both of its variations; the familial (familial interstitial fibrosis; FIP) or the sporadic IPF (Kropski et al., 2015) through mutations or SNPs in a variety of genes including surfactant proteins (Nogee et al., 2001; Wang et al., 2009; Campo et al., 2014), telomere network (Armanios et al., 2007; Tsakiri et al., 2007; Alder et al., 2008; Alder et al., 2011; Fukuhara et al., 2013; Kropski et al., 2014; Alder et al., 2015; Cogan et al., 2015; Kropski et al., 2015; Kropski et al., 2017; Stock and Renzoni, 2021) and immune-modulators and inflammation (Korthagen et al., 2012; Ahn et al., 2011; O'Dwyer et al., 2013; Xu et al., 2017; Son et al., 2013; McDonough et al., 2019; Allen et al., 2017). Gene expression profiles of IPF patients revealed a subgroup in which the calcium signaling pathway was upregulated (Wang et al., 2017; Zhang et al., 2021).

Mitochondrial dysfunction in IPF was reported by a number of studies (Bueno et al., 2015; Zank et al., 2018; Bueno et al., 2020; Luis-García et al., 2021; Liu et al., 2023; Mercader-Barceló et al., 2023). Bueno et al. (2015) reported “marked accumulation of dysmorphic and dysfunction mitochondria” in the alveolar type II cells of IPF patients. Subsequent reports demonstrated mitochondrial dysfunction in pulmonary fibroblasts (Luis-García et al., 2021) and lung resident mesenchymal stem cells (Mercader-Barceló et al., 2023) of IPF patients.

Intracellular calcium [Ca^++^]_i_ is one of the major signal transduction module in a variety of cell types. It plays a pivotal role in diverse stimulus-response coupling leading to the assignment of an appropriate response to a particular stimulus (Hallett and Hallett, 1989). Calcium signals are defined by their amplitude (amplitude-modulated; AM) and/or frequency (frequency-modulated; FM). Abnormal calcium oscillations have been seen in fibroblasts isolated from PF patients (Janssen et al., 2015). Furthermore, the profibrotic effect of transforming growth factor-β (TGF-β) was shown to be mediated by intracellular calcium oscillations (Mukherjee et al., 2012), and the latter was demonstrated to be sufficient to “modulate” extracellular matrix gene expression in the absence of any other stimulus (Mukherjee et al., 2017). Moreover, the profibrotic effect of the chemokine ligand 18 (CCL18), which has been reported to exist at high levels in lung fibrosis patients (Tiev et al., 2011), was demonstrated to be calcium dependent (Luzina et al., 2006). In addition, Mukherjee et al. have demonstrated that the antifibrotic effect of prostaglandin E_2_ was associated with interference with calcium-signaling and CaMK-II activation (Mukherjee et al., 2019). An inappropriate calcium response has been linked to IPF through the G protein-coupled calcium sensing receptor (CaSR) (Wolffs et al., 2019). Schuliga et al. have demonstrated the association of the calcium binding protein annexin A2 with regional fibrosis in IPF patients and linked it to cytokine production and fibrogenic gene expression (Schuliga et al., 2017).

We have recently identified patients of bronchocentric interstitial fibrosis with impaired oxygen transfer and chronic type 2 respiratory failure (Al-Mutairy et al., 2019). The disease is associated with a hypomorphic mutation in the gene for the calcium binding protein S100A3 and a novel truncating mutation in the gene for the S100A13 protein (Al-Mutairy et al., 2019). We demonstrated aberrant intracellular calcium homeostasis in the patient-derived fibroblasts, together with mitochondrial dysregulation and differential expression of the extracellular matrix proteins (ECM) (Al-Mutairy et al., 2019). Here we extend our finding to show that we can restore calcium homeostasis and rescue mitochondrial responses to levels comparable to control cells, by either transfecting patients-derived cells with wild-type genes or by treating these cells with extracellular recombinant wild-type proteins. We also demonstrate that transfecting control “normal” human bronchial epithelial cells with the mutant constructs causes calcium and mitochondrial anomalies similar to those found in patient derived cells. Finally, we show that patient-derived cells and human bronchial epithelial cells transfected with mutant *S100A3* and/or *S100A13* constitutively secrete the cytokines IL-6, IL-8 and MCP-1.

Our novel data demonstrate a causative effect of the mutant *S100A3* and *S100A13* on calcium homeostasis and suggest the potentially beneficial use of extracellular treatment in this subgroup of patients with the recombinant wild type proteins. Our data further suggest the necessity to develop calcium-based therapy for this so far untreatable disease.

## Materials and methods

The study was approved by the Research Advisory Council and the IRB of King Faisal Specialist Hospital and Research Centre (KFSH&RC-RAC 2120 009).

### Cell isolation and preparation

Skin biopsies were collected from patients and controls according to IRB-approved protocols. Fibroblasts were isolated and cultured as previously described (Shamseldin et al., 2017). Lung cell line (BEAS-2B ATCC^R^ CRL-9609) was purchased from ATCC (American Type Culture Collection, VA, United States). Cells were cultured in a humidified atmosphere of 5% CO_2_ at a temperature of 37°C in DMEM medium supplemented with 10% bovine calf serum, 2 mM glutamine, 2 mM sodium pyruvate, and 50 mg/L gentamicin (all from Invitrogen, Carlsbad, CA). Cell viability and cell numbers were validated using the MTS [3-(4,5-dimethyl-2-yl)-5-(3-carboxymethoxyphenyl)-2-(4-sulfophenyl)-2H-tetrazolium, inner salt)-based CellTiter Aqueous™ assays (Promega, Madison, WI) per manufacturer’s recommendations. Unless otherwise stated each experiment was done in triplicates with three different cell preparations. Patients’ cells were isolated from two affected individuals of two different families. Control cells were isolated from two unaffected healthy donors.

### S100A3 and S100A13 expression

The coding sequences of wild-type and mutant S100A3 were codon-optimized for mammalian expression. The sequences were preceded by the consensus Kozak sequence and followed by sequences encoding a 15 amino acid-long flexible linker (GGGGS)_3_ and the HA tag (YPYDVPDYA). The resulting fusion proteins were 125 amino acids long. The constructs were artificially synthesized (GenScript, Piscataway, NJ), cloned downstream of the CMV promoter into the pVQAd5CMVK-NpA plasmid (ViraQuest, North Liberty, IA) as previously described (Kopach et al., 2014), and the sequences were confirmed by automated bi-directional sequencing. S100A13 coding sequences of wild-type and mutant S100A13 were amplified by PCR using the forward primer ATG​GCA​GCA​GAA​CCA​CTG​AC and the reverse primer CTT​CTT​CCT​GAT​CTT​CAG, cloned into the empty coding region of gWIZ Blank Mammalian Expression Vector (Cat.# P000200, Genlantis, Gene Therapy Systems, Inc.) downstream from the CMV promoter. The generated S100A13 sequence was MYC-Tagged (EQKLISEEDL) and the resulting fusion proteins were 123 amino acids long. All cloned sequences were confirmed by DNA sequencing. In experiments, we overexpressed C-terminally HA-tagged mutant or wild-type S100A3 and/or Myc-tagged S100A13 in fibroblasts isolated from “normal” adult, patients and BEAS-2B cell line. A vector containing red fluorescent protein (RFP) was used in the transfection mixture as a marker for positive transfection. Parallel experiments were carried out in which cells were transfected with RFP-containing vector only.

### Transfection of cultured cells

Fibroblasts and BEAS-2B cells were seeded on coverslips overnight and were subsequently transfected using Lipofectamine™ 3000 Kit (Cat. # L3000-015, Lot # 2041107 from Invitrogen, ThermoFisher Scientific, United States) according to manufacturer’s instruction. Transfection efficiency was estimated by co-transfection with RFP. Experiments were performed only on cells exhibiting 70%–80% transfection efficiency 24 h post transfection.

### Flow cytometry analyses of mitochondria

Trypsinized cells (0.5–1.0 × 10^6^) cell/mL were labelled with Mitrotracker Green FM (Cat # M-7514, Lot # 3281-2, Molecular Probes™, United States) for 15 min at 37°C. Washed cells (PBS, pH 7.2) were re-suspended in culture medium and intensity measurements were taken using FACSCalibar flow cytometer (BD Bioscience).

### Intracellular calcium and mitochondrial integrity measurements

Intracellular calcium measurements were performed on patient fibroblasts, control fibroblasts and BEAS-2B ATCC^R^ CRL-9609 as described previously (Al-Mutairy et al., 2019). Receptor mediated changes in intracellular fluorescence intensity in response to Bradykinin (BK; 50 µM) and Ionomycin (2 µM) were followed using Zeiss LSM 510 META laser scanning confocal system (Carl Zeiss MicroImaging, GmbH, Germany). Mitochondrial mass was assessed using Mito Tracker^®^Green (2 µM, Invitrogen™ Molecular Probes™, United States), and viewed under Zeiss Yokogawa Spinning Disk confocal system (Carl Zeiss MicroImaging, GmbH, Germany). JC-1 (5,5′,6,6′-tetrachloro-1,1′3,3′-tetraethylbenzimidazolylcarbocyanine iodide) was purchased from ThermoFisher. JC-1 is a cationic dye that accumulates in the mitochondrial matrix (Di Lisa et al., 1995; Chazotte, 2011; Al-Zubaidi et al., 2019). The accumulated dye exists in two forms; monomeric (green) and aggregate (J aggregate, red), depending on the mitochondrial membrane potential (*∆Ψ*_*m*_). The fluorescence ratio of red/green increases with increased polarization of the *∆Ψ*_*m*_. Cells were incubated with 5 µM of the dye at 37°C for 30 min and fluorescence intensity was measured using Zeiss Yokogawa Spinning Disk confocal system (Carl Zeiss MicroImaging, GmbH, Germany).

### Immunofluorescence, fluorescence labeling and Western blotting

Primary antibodies to S100A3, S100A13 and fluorescein-conjugated secondary anti-rabbit IgG were purchased from Abcam (Abcam; Abdulla Fouad Medical Supplies, Dammam, Saudi Arabia) and ThermoFisher Scientific (ThermoFisher Scientific, Waltham, MA, United States), respectively. The antibodies were used according to the manufacturers’ instructions. Recombinant S100A3 and S100A13 proteins were obtained from Abnova and Abcam (Taipei, Taiwan and Cambridge, United Kingdom, respectively). Western blots were performed as essentially described previously (Al-Mutairy et al., 2019). Anti-tag antibodies were purchased from Roche (HA-Tag, Cat# 11608200) and from Invitrogen (Myc-Tag, Cat# 460709) and used at 1:1000 dilution.

### Measurement of inflammatory mediators

Inflammatory mediators were measured using Milliplex Map (xMAP^®^ technology, Luminex, TX,United States) according to the manufacturer’s instruction. MILLIPLEX kits were purchased from Millipore (Cat# HCYTOMAG-60K-41, Lot # 3158918). Measurements were performed in triplicates as described previously (Al-Anazi et al., 2018).

### Statistical analyses

ANOVA with Dunnett’s multiple comparison test was used to measure statistical significance using Prism (GraphPad, La Jolla, CA, United States). Unpaired two-tailed t-test was used when appropriate. A *p*-value ≤ 0.05 was considered significant.

## Results

### S100A3 and S100A13 mutations interfere with receptor-mediated calcium responses and release of intracellular calcium stores

**FIGURE 1:**
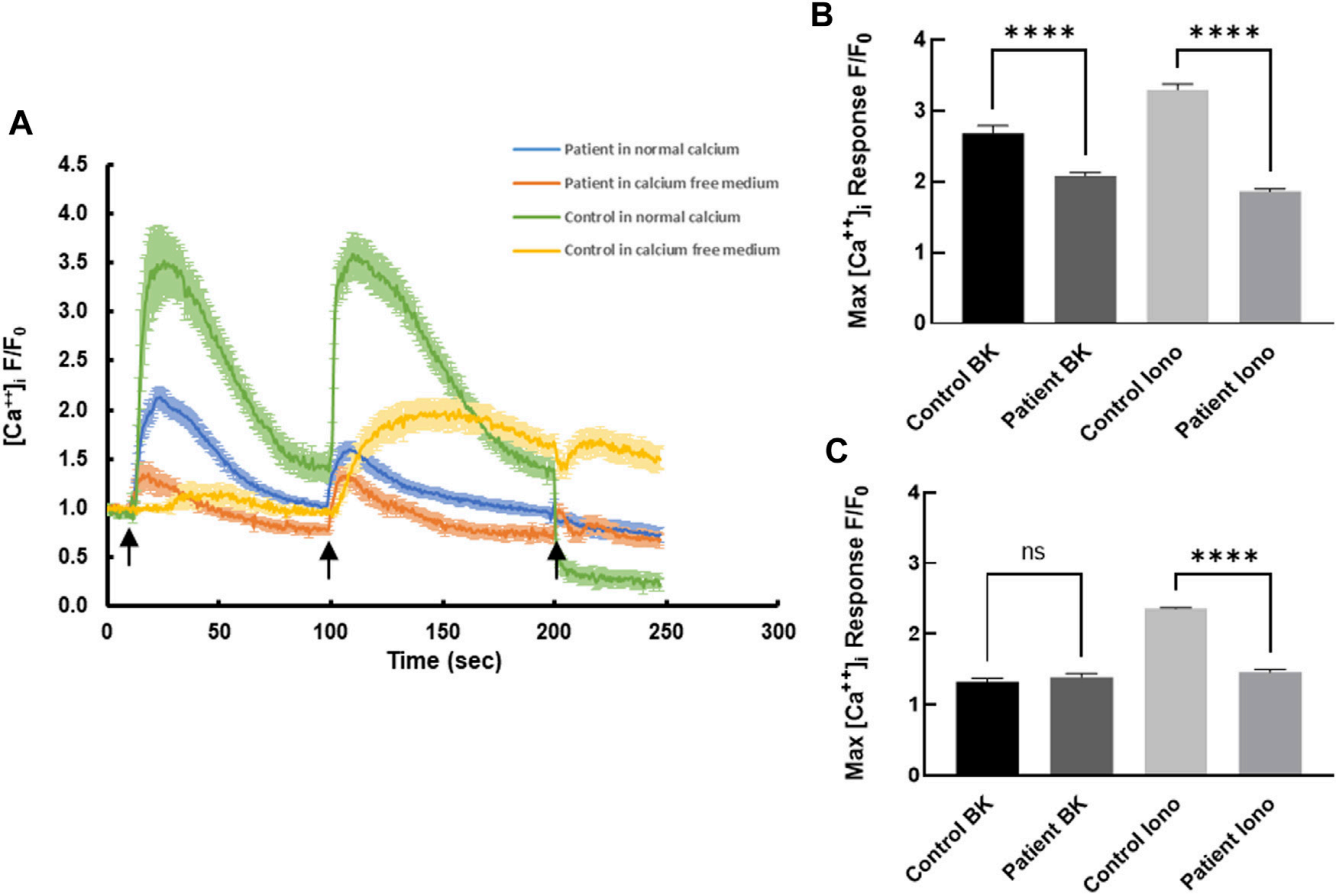
Intracellular calcium responses in patient- and healthy control-derived skin fibroblasts. **(A)** Kinetics of bradykinin (BK)- (50 µM) induced (first arrow) and ionomycin- (2 µM) induced (second arrow) calcium changes in fibroblasts isolated from healthy controls (green) and patients (blue) in normal Krebs-HEPES buffer and in calcium free Krebs-HEPES (yellow and red, respectively). EGTA (1 mM) was added at the third arrow. **(B)** Peak intracellular calcium levels in response to BK (50 µM) stimulation in patient and control cells in the presence of extracellular calcium. **(C)** Peak intracellular calcium levels in response to ionomycin (2 µM) stimulation in patient and control cells in the absence of extracellular calcium. **** indicates *p < 0.0001* ns is not significant. The data are expressed as mean ± SEM and are representative of at least three independent experiments performed in triplicates with cells isolated from two different patients belonging to two different families and two different controls.

Receptor-mediated intracellular calcium transients in response to bradykinin were measured using live cell confocal imaging. Treatment of healthy control-derived skin fibroblasts with bradykinin (BK, 50 µM) evoked a transient rise in intracellular calcium reaching a maximum of 2.68 ± 0.11 fold (normalized fold increase compared with baseline) before declining back to the pre-stimulatory levels (Figure 1A). Subsequent treatment with the calcium ionophore ionomycin (2 µM) evoked a second transient reaching a maximum of 3.30 ± 0.08 fold with slow return to pre-stimulatory levels. Compared with control cells, the bradykinin-evoked and ionomycin-evoked calcium signals were blunted in patient-derived skin fibroblasts rising to a maximum of 2.08 ± 0.05 fold baseline (Figure 1B) and 1.89 ± 0.04 fold baseline (*p* < 0.0001; Figure 1B), respectively. The significant difference between bradykinin mediated calcium transients in patients and controls was completely nullified in the absence of extracellular calcium, whereas the ionophore-induced calcium transients persisted even in the absence of extracellular calcium (Figure 1C), suggesting the S100A3/S100A13 mutations reduce both extracellular calcium influx and intracellular calcium release.

**FIGURE 2:**
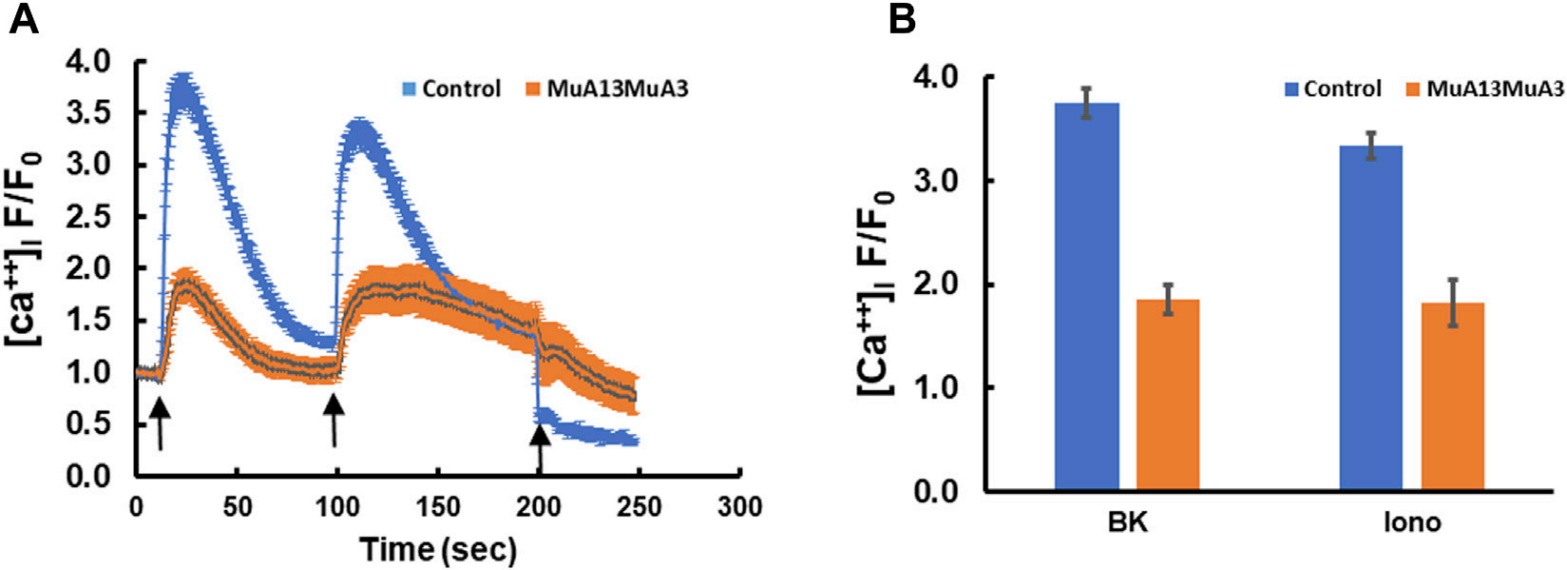
Intracellular calcium responses in double transfected (mutant *S100A3+*mutant *S100A13*) control cells. **(A)** Kinetics of BK- (50 µM) induced (first arrow) and ionomycin- (2 µM) induced (second arrow) in human skin fibroblasts transfected with mutant *S100A3* and mutant *S100A13* (red) and control skin fibroblasts transfected with RFP (Blue). EGTA (1 mM) was added at the third arrow. **(B)** Peak intracellular calcium levels in response to BK (50 µM) and ionomycin (2 µM) in control RFP transfected (blue) and control mutant *S100A3* and mutant *S100A13* transfected (red) fibroblasts. The data are expressed as mean ± SEM and are representative of at least three independent experiments performed in triplicates.

Transfection of plasmids expressing the mutant *S100A3* and mutant *S100A13* in fibroblasts isolated from healthy normal adult human skin blunted BK (50 µM) and ionomycin (2 µM)-evoked calcium transients, suggesting a direct effect of the mutant proteins on calcium homeostasis (Figures 2A, B). Whereas, control cells transfected with RFP-expressing vector only exhibited little change in either bradykinin or ionomycin-induced calcium transients, fibroblasts co-transfection with genes for mutant S100A3 and S100A13 exhibited calcium transients that were reduced by half compared with control cells (1.86 ± 0.14 fold vs. 3.74 ± 0.14 fold for BK-evoked and 1.82 ± 0.22 fold from the control 3.34 ± 0.12 fold for ionomycin-evoked calcium transients.

### Restoration of calcium transients by transfection of patients’ cells with WT *S100A3*, *S100A13* or treatment with recombinant proteins extracellularly

**FIGURE 3:**
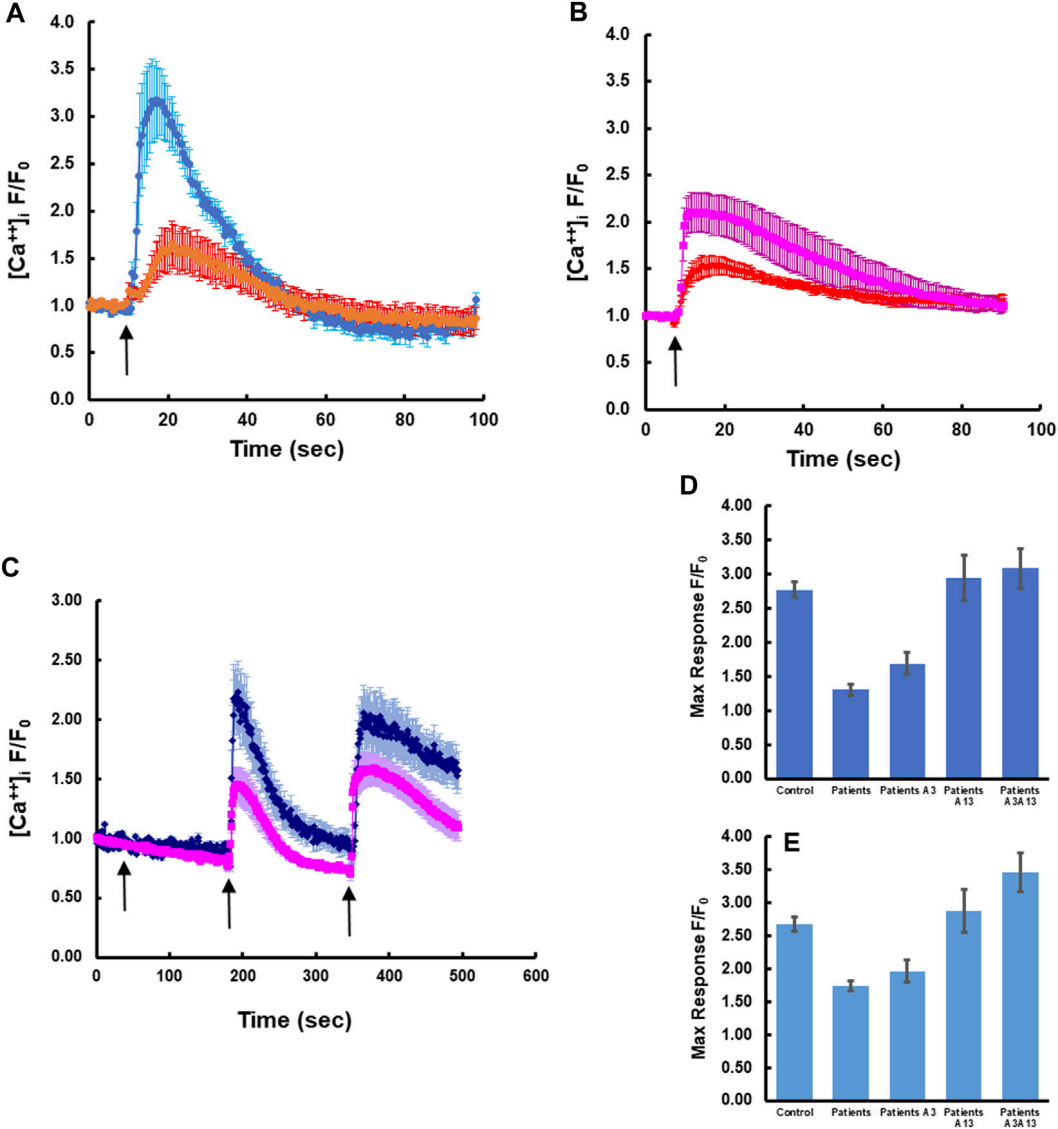
Rectifying the intracellular calcium signaling in patients-derived cells by transfection with wild type transcripts or treatment with extracellular recombinant proteins. **(A)** Patient-derived fibroblasts transfected with wild type *S100A13* (blue) compared to un-transfected cells (red). **(B)** Patient-derived fibroblasts transfected with wild type *S100A3* (pink) compared to un-transfected cells (red). **(C)** Treatment of patient-derived cells with the recombinant proteins S100A13 (0.5 μg/mL) and S100A3 (0.5 μg/mL) restores BK-induced (50 µM) and ionomycin-induced (2 µM) calcium transients (blue trace) compared to untreated patient-derived cells (pink). **(D)** Peak calcium levels induced in patient cells treated with the recombinant proteins in response to BK and **(E)** Peak calcium levels induced in patient cells treated with the recombinant proteins in response to ionomycin. The data are expressed as mean ± SEM and are representative of at least three independent experiments performed in triplicates with cells isolated from two different patients belonging to two different families.

To determine whether expression of wild-type S100A3 and/or S100A13 could restore normal calcium signaling in skin fibroblasts isolated from patients carrying the S100A3/S100A13 mutations, we analyzed calcium signaling in patient cells transfected with expression vectors containing wild type *S100A3* and/or *S100A13* sequence. We found that in these cells, responses to BK and Ionomycin were restored by either of the two transfected WT genes to levels comparable to that of control cells (Figures 3A, B). Because transfection of patients’ cells was cumbersome and with variable toxicity, we ran parallel experiments using recombinant S100A3 and S100A13 proteins. We found that exposure of patient’s cells to the recombinant proteins evoked similar calcium effects to transfection with the wild-type transcripts. Interestingly treatment of patient cells with recombinant S100A3 (500 ng/mL) for 5 min prior to stimulation with BK caused a small but non-significant increase in BK response (1.69 ± 0.17 compared to 1.30 ± 0.08 normalized fold increase for treated and non-treated patients’ cells, respectively). Recombinant S100A13 (500 ng/mL) on the other hand, significantly restored the response compared to untreated cells (2.94 ± 0.33 fold compared to 1.30 ± 0.08, *p < 0.0001*) for treated and untreated, respectively. The response of the S100A13 treated cells was similar to that of cells isolated from normal control (2.77 ± 0.11). Combined treatment of patients’ cells with recombinant S100A3 and S100A13 (500 ng/mL each) restored both bradykinin-mediated and ionomycin-mediated calcium transients to levels that were comparable to control cells responses. The recombinant proteins had no apparent direct effect on the resting calcium levels (Figure 3C).

### Restoring the calcium responses in patient’s cells rectifies mitochondrial anomalies

**FIGURE 4:**
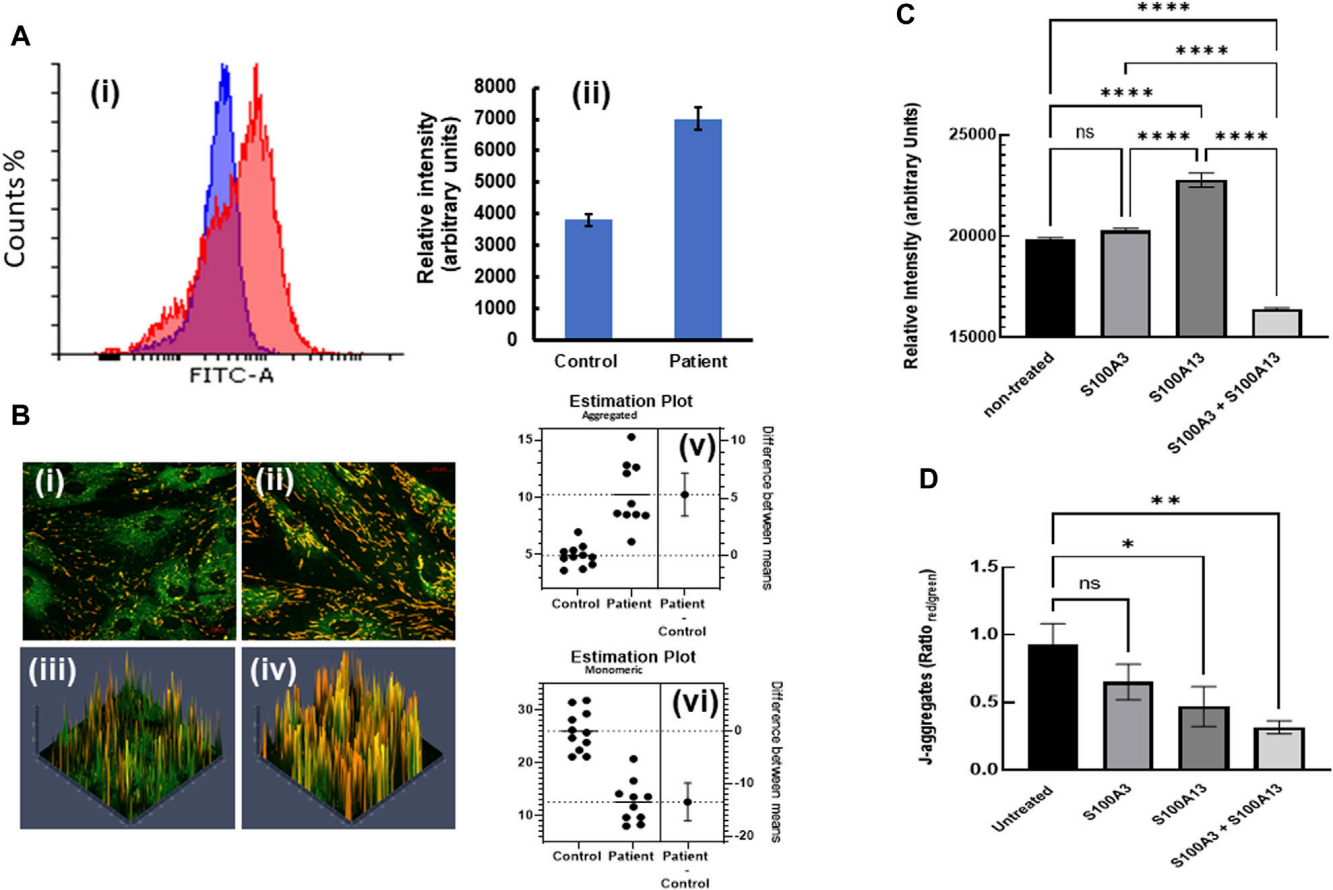
Restoring mitochondrial mass and hyperpolarization by treatment with extracellular recombinant S100A3 and S100A13 proteins. **(A)** Flow cytometry histograms (i) illustrating increased mitochondrial staining (increased mass) with MitoTracker Green FM (1 µM) in patient (red) vs. control (blue) cells with relative intensities (mean ± SEM) shown in (ii). **(B)** Confocal fluorescence micrographs of JC-1 labelled control- (i) patient-derived cells (ii) and corresponding 3D intensity maps color coded so that J-aggregates are red and monomeric JC-1 is green (iii and iv, respectively). Estimation plots showing difference between means of J-aggregates (upper panel) and monomeric JC-1 (lower panel) in control and patient cells are displayed in v and vi, respectively. **(C)** Effects of treatment of patient-derived cells with recombinant proteins (0.5 μg/mL, 24 h) on mitochondrial mass. **(D)** Effects of treatment of patient-derived cells with recombinant proteins (0.5 μg/mL, 24 h) on mitochondrial hyperpolarization as measured by J-aggregate accumulation. *p*-values < 0.0001 (****), 0.007 (**), 0.04 (*), ns is not significant. The data are expressed as mean ± SEM and are representative of at least three independent experiments performed in triplicates with cells isolated from two different patients belonging to two different families and two different controls.

This digenic inheritance was also associated with mitochondrial anomalies (Al-Mutairy et al., 2019), in which patient-derived cells exhibited a significantly higher mitochondrial mass compared to normal control cells (Figure 4A). Here we extended our study by measuring the mitochondrial membrane potential in both control and patient-derived cells through investigating the spectral characteristics of JC-1 (*5, 5′, 6, 6′-tetrachloro-1,1′,3,3′-tetraethylbenzimidazolylcarbocyanide iodide)*-labelled cells. We found that patient-derived cells displayed significantly more J-aggregates (*p < 0.0001*) than control cells (Figure 4B). Conversely, JC-1 monomer accumulation was significantly higher in control (*p < 0.0001*) than in patient cells (Figure 4B), suggesting more polarized mitochondria, thus higher *∆Ψ*_*m*_ in patient cells compared to control. Moreover, treatment of patient cells with extracellular S100A3 and S100A13 together (500 ng/mL each for 24 h at 37°C) reduced the mitochondrial mass significantly (Figure 4C). However, whereas treatment with extracellular S100A3 had no apparent effect on the mitochondrial mass, treatment with extracellular S100A13 significantly increased mitochondrial mass (*p < 0.0001*). Furthermore, treatment of patient’s cells with S100A3 had no apparent effect on *∆Ψ*_*m*_, whereas treatment with S100A13 significantly reduced J-aggregates accumulation which is consistent with reduced *∆Ψ*_*m*_ (*p = 0.0443*, Figure 4D). Combined treatment with both proteins extracellularly displayed an additive reduction in *∆Ψ*_*m*_ compared to treatment with either protein alone (*p < 0.007*), further confirming the more complex interactions between S100A3/S100A13 and mitochondrial *∆Ψ*_*m*_ (Figure 4D).

### Constitutive secretion of IL-1α, IL-8, IL-6 and MCP-1 in patient-derived fibroblasts

**FIGURE 5:**
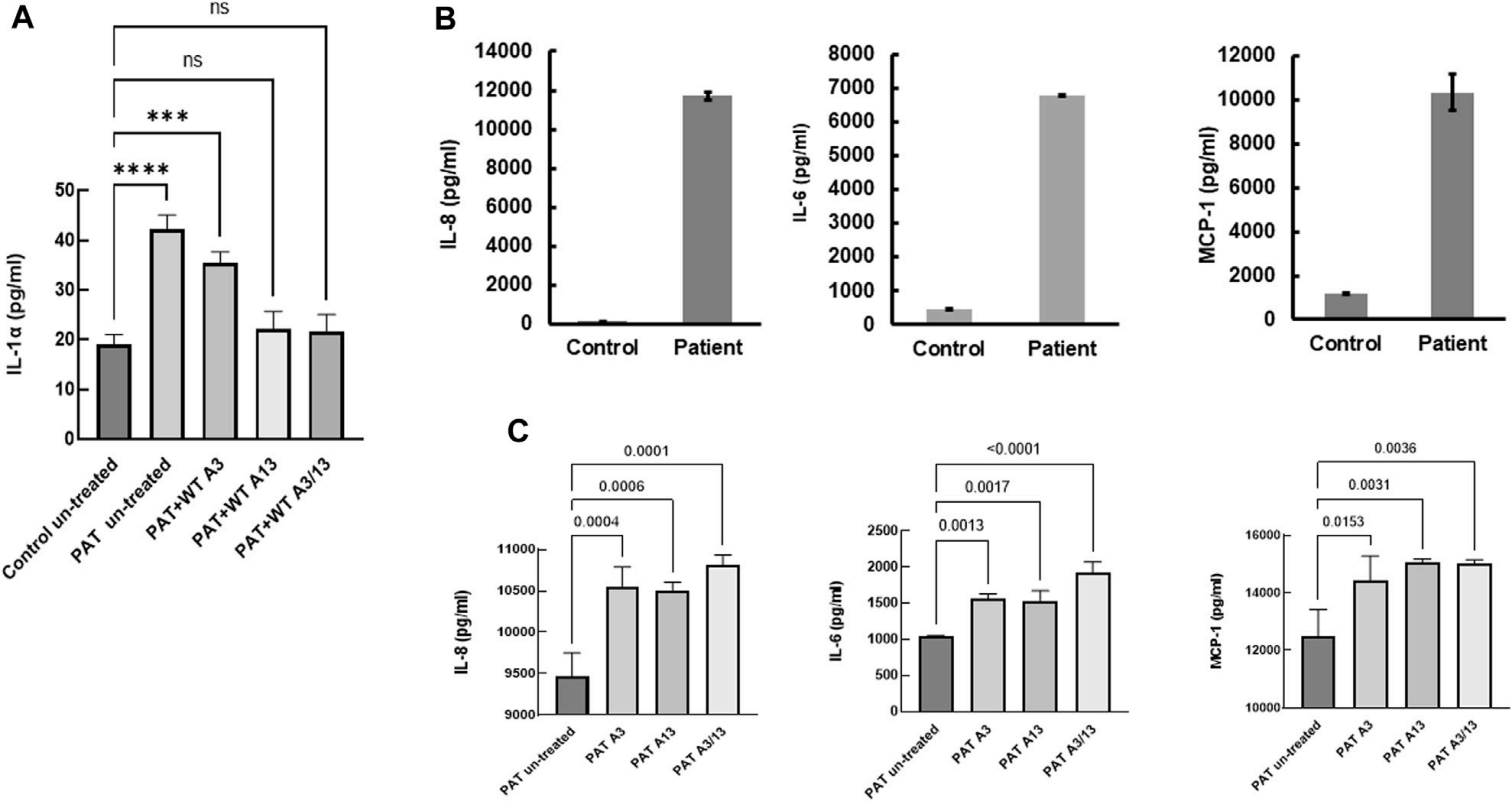
Constitutive secretion of inflammatory cytokines in patients-derived and healthy control-derived fibroblasts. **(A)** Release of IL-1α in patients-derived fibroblasts (PAT un-treated) compared to control (Control untreated), the effect of treatment with extracellular recombinant S100A3 (0.5 μg/mL, PAT + WT A3), the effect of treatment with extracellular recombinant S100A13 (0.5 μg/mL, PAT + WT A13) and the effect of treatment with extracellular recombinant S100A3 (0.5 μg/mL) and S100A13 (0.5 μg/mL) together (PAT + WT A3/A13). Treatment was for 18hrs at 37°C in complete DMEM media as described in Methods. **(B)** Constitutive secretion of IL-8, IL-6 and MCP-1 in patient-derived fibroblasts (Patient) compared to secretion in control-derived cells (Control). **(C)** Effects of treatment of patient-derived fibroblasts with extracellular recombinant S100A3 (0.5 μg/mL, PAT A3) and S100A13 (0.5 μg/mL, PAT A13) together (PAT + WT A3/A13). Treatment was for 18 h at 37°C in complete DMEM media as described in Methods. *p values* are indicated on the horizontal bars. Data are from representative experiments performed in triplicates with cells isolated from two different patients belonging to two different families and two different controls.

The role of inflammatory mediators in the pathogenesis of lung fibrosis has been realized for a number of years (Sama and Norris, 2013; Kobayashi et al., 2015; Luzina et al., 2015; She et al., 2021). Since aberrant calcium signaling is pivotal to inflammatory responses (Sama and Norris, 2013) and S100A13 has been implicated in the constitutive secretion of IL-1α (Mohan and Yu, 2011), we compared constitutive cytokine secretion in patient-derived and controlled cells. Constitutive levels of IL-1α secretion were over 2-fold higher in patient-derived cells compared to control (42.2 ± 1.7 pg/mL vs. 19 ± 1.1 pg/mL, *p < 0.0001*, Figure 5A). Treatment with extracellular recombinant wild-type S100A13 reduced this to levels comparable to those found in control fibroblasts (Figure 5A); whereas S100A3 had no apparent effect. Similarly, secretion of proinflammatory cytokines IL-6, IL-8 and MCP-1 were all substantially higher in patient-derived than controlled cells (6775 ± 29 pg/mL, 11,716 ± 184 pg/mL and 10,331 ± 830 pg/mL compared to control samples of 445 ± 14 pg/mL, 147 ± 7 pg/mL and 1201 ± 33 pg/mL for IL-6, IL-8 and MCP-1, respectively; Figure 5B). Contrary to IL-1α, attempts to modulate the levels of secreted IL-6, IL-8 and MCP-1 with extracellular treatment of patient’s cells with the recombinant proteins failed to reduce the levels of these cytokines (Figure 5C).

### Effect of S100A3 and *S100A13* mutations on bronchus epithelial cells

**FIGURE 6:**
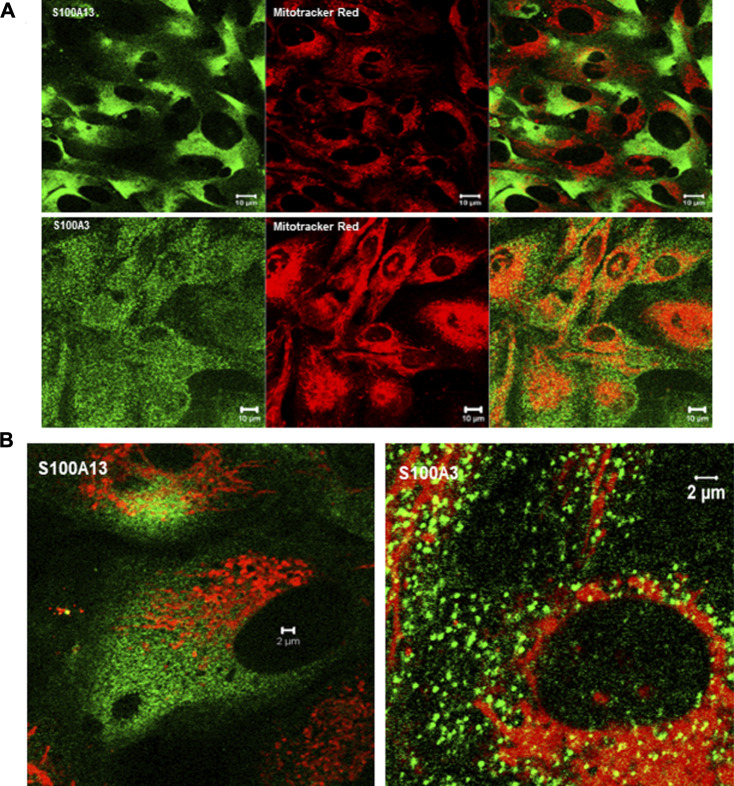
Expression of S100A3 and S100A13 in bronchus epithelial cell line BEAS-2B cells. **(A)** Confocal immunofluorescence micrographs illustrating the expression of S100A3 and S100A13 (green) and mitochondria (red) and the corresponding superimposed images. Scale bar is 10 µm. **(B)** High resolution confocal immunofluorescence micrographs showing the distribution of S100A3 (green) superimposed on mitochondrial staining (Mitotracker red) compared to the distribution of S100A13 (green) and Mitotracker red). Scale bars are 2 µm.

**FIGURE 7:**
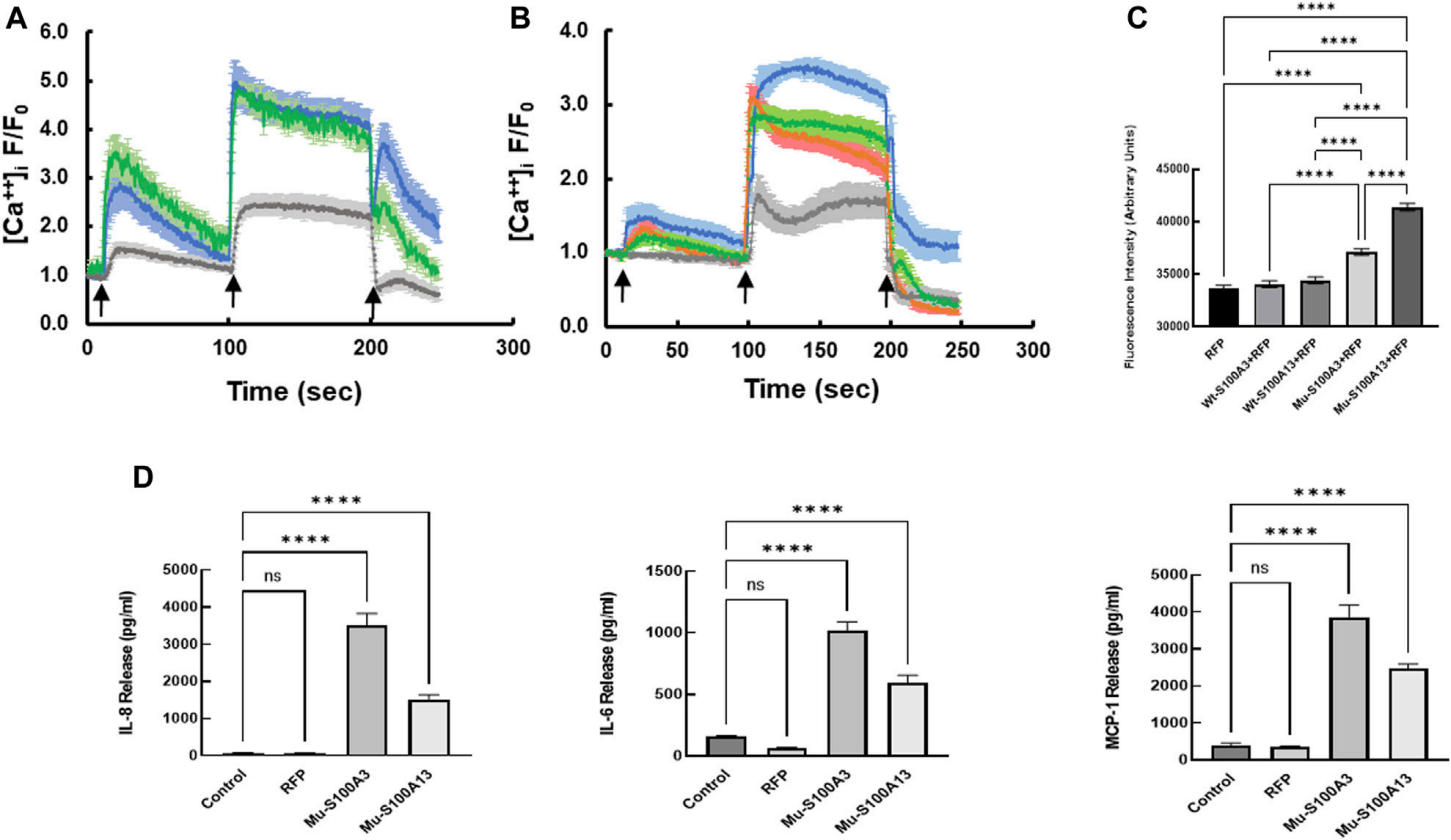
Effect of mutant S100A3 and S100A13 on intracellular calcium, mitochondria and cytokine secretion in bronchus epithelial cell line BEAS-2B cells. **(A)** Kinetics of calcium changes in response to BK (50 μM, first arrow) and ionomycin (2 μM, second arrow) in un-transfected (blue), wild type *S100A3-*transfected (green), and mutant *S100A3*-transfected (gray) bronchus epithelial cells. EGTA (1 mM) was added at the third arrow. **(B)** Kinetics of calcium changes in response to BK (50 μM, first arrow) and ionomycin (2 μM, second arrow) in un-transfected (blue), wild type *S100A13-*transfected (green), and mutant *S100A13*-transfected (gray) and RFP-transfected (red) bronchus epithelial cells. EGTA (1 mM) was added at the third arrow. **(C)** Mutant but not wild type S100A3 and S100A13 cause increased mass of mitochondria as measured by Mitotracker Green AM staining. RFP is cells transfected with red fluorescent protein, Wt-S100A3+RFP is cells transfected with both wild type S100A3 and RFP, Wt-S100A13+RFP is cells transfected with S100A13 together with RFP, Mu-S100A3+RFP is cells transfected with mutant S100A3 together with RFP and Mu-S100A13+RFP is cells transfected with mutant S100A13 together with RFP. **** indicates *p* value of <0.0001. **(D)** Mutant S100A3 (Mu-S100A3) and Mutant S100A13 (Mu-S100A13) induce IL-8, IL-6 and MCP-1 release in transfected bronchus epithelial cell line BEAS-2B. Control is un-transfected cells and RFP is cells transfected with plasmid carrying RFP only. **** indicates *p value* < 0.0001. Data are performed in triplicates and are representative of three independent experiments.

To extend this work to lung pathology, we analyzed the effect of mutant S100A3 and S100A13 on calcium handling in the bronchus epithelial cell line BEAS-2B cells (ATCC^®^ CRL-9609^™^). Immunofluorescence analysis of endogenous S100A3 and S100 A13 in resting BEAS-2B cells revealed distinct distribution patterns for the two proteins. S100A3 exhibited a punctate cytosolic distribution throughout the cytosol (Figure 6A); whereas S100A13 was essentially in areas lacking mitochondria (Figure 6B). Transfection with either mutant *S100A3* and/or mutant *S100A13* in these cells caused a significant reduction in the bradykinin-induced and ionomycin-induced calcium transients (Figures 7A, B), confirming a direct effect of the mutations on calcium homeostasis and implicating S100A3 and S100A13 in normal cellular calcium responses. The effect of the mutations on the mitochondria of transfected BEAS-2B cells was similar to that seen in patient-derived skin fibroblasts, confirming the profound effect of the S100A3 and S100A13 on mitochondrial activity (Figure 7C).

The effect of mutant S100A3 and S100A13 on cytokine expression was extended to bronchial epithelial cells by transfecting the BEAS-2B cell line with vectors expressing mutant *S100A3* or *S100A13*. Under such conditions, both mutant transcripts caused significant cytokine secretion compared to un-transfected and red fluorescent protein (RFP)-transfected controls (Figure 7D).

## Discussion

We have previously described an atypical and early-onset form of lung fibrosis which segregates in patients carrying digenic mutations in the calcium-binding proteins S100A3 and S100A13 (Al-Mutairy et al., 2019). Here we extend our finding to show a direct effect of the mutations on calcium homeostasis. We provide evidence to show that: 1) expressing/overexpressing mutant proteins in normal cells caused similar aberrant calcium responses to receptor-mediated calcium transients and ionophore-induced calcium release as seen in patient-derived cells, 2) transfecting patient-derived cells with the wild-type genes normalizes the calcium responses and mitochondrial dysfunction, and 3) exposing patient-derived cells to extracellular recombinant S100A3 and S100A13 proteins normalize calcium and mitochondrial anomalies suggesting a possible novel therapeutic strategy for treating patients with this genetic abnormality.

We present data demonstrating differences in mitochondria biogenesis of patient derived cells and cells transfected with the mutant transcripts compared to control. We show that patient cells had greater mitochondrial mass and more hyperpolarized mitochondria than control. The combination of S100A13 and S100A3 reduced both mitochondrial density and polarization toward levels seen in control cells. In contrast S100A13 alone increased mitochondrial density while reducing polarization. On the other hand, S100A3 alone did not alter either mitochondrial density or polarization. Persistent mitochondrial hyperpolarization has been linked to ATP depletion, cytoplasmic alkalinization and increased reactive oxygen species (ROS) production (Perl et al., 2004). Although we have not measured ATP production or cytoplasmic alkalinization, ROS production was found to be elevated in patient-derived cells (Al-Mutairy et al., 2019).

The observed increase in mitochondrial biogenesis may involve the generation of new mitochondria and/or impaired mitophagy; the quality control mechanism that selectively remove damaged or surplus mitochondria. The link between the two is dynamic and essential for mitochondrial homeostasis (Popov, 2020). When damaged mitochondria are identified, specific signaling pathways are activated. For example, PTEN-induced kinase 1 (PINK1) (Poole et al., 2008; Springer and Kahle, 2011) accumulates on their outer membrane and recruits Parkin, a ubiquitin ligase, which tags the damaged mitochondria for degradation. Simultaneously, transcription factors such as NRF1 and NRF2 (Gureev et al., 2019; Lu et al., 2020; Gumeni et al., 2021) may be activated, which promote mitochondrial biogenesis. The question as to whether such signaling is still intact in our patients’ samples is yet to be established.

Since S100A13 is intimately linked to IL-1α release through the non-classical pathway of secretion (Mohan and Yu, 2011), the possibility existed that the absence of S100A13 may also lead to IL-1α dysregulation. We measured IL-1α in conditioned media from patient-derived cells and found it to be statistically significantly different from that obtained in healthy control-derived cells. The role of IL-1α as an inflammation-inducing danger signal is well documented (Kaneko et al., 2019). Its dysregulation affects the whole IL-1 signaling pathway/machinery that includes IL-1β, IL-18, IL-33, IL-36α, IL-36β, IL-36γ, IL-37, IL-38, IL-1Ra and IL-36Ra (Kaneko et al., 2019; Dagvadorj et al., 2021). Moreover, since increased peripheral blood levels of IL-6 and IL-8 have been associated with “exacerbated IPF” in comparison to stable IPF (Papiris et al., 2018; She et al., 2021), we measured the levels of IL-6 and IL-8 as well as MCP-1 in patient-derived cells and control cells transfected with the mutant *S100A3* and *S100A13*. We demonstrate significantly higher levels of inflammatory cytokines IL-8, IL-6 and MCP-1, in conditioned media isolated from patient-derived cells and in control cells transfected with the mutant *S100A3* and *S100A13*. Although cytokine levels have been implicated in pulmonary fibrosis (Hartl et al., 2005; Kobayashi et al., 2015; Papiris et al., 2018; Yang et al., 2018; She et al., 2021), the source of these cytokines has been attributed to pulmonary macrophages M1/M2 activation network (Liu et al., 2019). Here we demonstrate that in the absence of macrophages or any other cell type, the patient’s fibroblasts alone were able to constitutively produce inflammatory cytokines incriminating *S100A3/S100A13* mutations directly in the observed cytokine secretion. This was further supported by our finding that transfection with the mutations in normal epithelial cells evoked similar constitutive release of the reported cytokines.

It is noteworthy that persistent mitochondrial hyperpolarization was reported to tip the balance towards necrosis rather than apoptosis in patients of systemic lupus erythematosus (SLE). It is, therefore, possible to tentatively speculate that necrosis may significantly contribute to the inflammatory process observed in pulmonary fibrosis patients. Whether the mitochondrial hyperpolarization seen in patient’s derived cells is associated with necrosis or not is yet to be investigated.

Although the pathogenicity associated with the co-inheritance of both mutations is clear, the question of whether either mutation alone can cause the disease is yet to be addressed. The mutation in *S100A3* is classified as a SNP and we previously hypothesized, based on its population frequency that by itself, it may not be pathogenic (Al-Mutairy et al., 2019). However, control cells transfected with the mutant *S100A3* displayed similar anomalous calcium and mitochondrial activities to those seen in patient-derived cells. This argues against a nonpathogenic effect of the SNP variant in S100A3 and suggests: 1) low pathogenicity in which the disease in the SNP (alone) carrying individuals is very mild and/or 2) a modifier effect invoked by the SNP. The mutation in *S100A13* is a frameshift mutation expected to result in a truncated form of the protein (Al-Mutairy et al., 2019) and subsequently its pathogenicity is understandable. In this case, treatment with the recombinant S100A13 protein or transfection with wild-type *S100A13* alone normalized the calcium changes in patient-derived cells to levels that were comparable to that of control cells. The interplay between these two calcium-binding proteins in bringing the fibrosis phenotype is yet to be determined.

In conclusion in this study, we provide direct evidence of a causative effect of the mutations on calcium (receptor mediated calcium signaling and calcium store responses) and mitochondrial homeostasis. Together our findings indicate that treatment of patients’ cells with recombinant S100A3 and S100A13 proteins is sufficient to normalize most of cellular responses, and may therefore suggest the use of these recombinant proteins in the treatment of this devastating disease.

## Data Availability

The original contributions presented in the study are included in the article/Supplementary Material, further inquiries can be directed to the corresponding author.
